# Protective mechanism of Xuebijing injection against heat stroke in rats

**DOI:** 10.3892/etm.2014.1639

**Published:** 2014-03-27

**Authors:** JUN JI, FEIHU ZHOU, HUI YUE, QING SONG

**Affiliations:** Department of Critical Care Medicine, Chinese People’s Liberation Army General Hospital, Beijing 100853, P.R. China

**Keywords:** heat stroke, Xuebijing injection, protection, cytokine, biochemical indicator, coagulation indicator

## Abstract

This study aimed to investigate the protective mechanism of Xuebijing injection (XBJ) against heat stroke (HS) in rats. Adult male Sprague Dawley rats were randomly divided into normal control (NC), normal saline-treated HS (NS-HS) and XBJ-treated HS (XBJ-HS) groups. At 47 and 57 min from the initiation of heat stress (42.5–43.5°C), the plasma levels of certain cytokines [interleukin (IL)-1β, IL-6 and tumor necrosis factor-α], biochemical indicators (creatinine, blood urea nitrogen, aspartate aminotransferase, alanine aminotransferase and alkaline phosphatase) and coagulation indicators (activated partial thromboplastin time, prothrombin time, fibrinogen degradation products and D-dimer) were detected, and microscopy of the liver tissue of the rats was conducted. At 47 and 57 min after the initiation of the heat stress, the levels of the cytokines, coagulation indicators and biochemical indicators in the NS-HS group were significantly higher than those in the NC group (P<0.05). In the NS-HS group, the levels of the aforementioned indices were significantly reduced compared with those in the NC and NS-HS groups (P<0.05). In the NS-HS group, serious liver cell congestion, nuclear swelling and central vein dilation were visible, along with the appearance of bubbles in the liver tissue. In the XBJ-HS group, only a small number of congestive liver cells were identified, with occasional nuclear swelling but no bubbles, which was similar to the observations in the NC group. Early intervention treatment of HS with XBJ is able to reduce the systemic inflammatory response and coagulation activity and decrease the tissue ischemia and injury degree, thus extending the survival time of rats with HS.

## Introduction

Heat stroke (HS) is a type of nerve damage caused by thermoregulatory dysfunction and excessive accumulation of body heat due to high temperature. The clinical symptoms mainly include high fever, no sweat and central nervous system disorders (1,2). HS is the most severe form of heat stress, with extensive damage to the body, and may lead to functional and morphological changes of numerous organs and systems. Once HS occurs, the mortality rate is as high as 63%, unless timely and proper treatment is received (3).

The pathophysiological process of HS is similar to that of severe sepsis. Cytokines may mediate the systemic inflammatory response, and play key roles in the process of HS (4). The uncontrolled systemic inflammatory response causes a cascade resulting in multiple organ dysfunction syndrome (MODS). Bouchama and Knochel (1) consider that the characteristic pathological and clinical manifestations of HS are the interaction results of complex physiological and biochemical mechanisms prior to body collapse, including thermoregulatory imbalance, enlargement of the acute-phase response and the expression of heat shock protein (HSP).

The traditional Chinese medicine preparation Xuebijing injection (XBJ) is produced and applied clinically. XBJ is able to antagonize bacterial toxins, reduce endotoxin levels, regulate immune and inflammatory mediators, improve microcirculation and protect vascular endothelial cells. It has been demonstrated that XBJ can significantly increase the survival rate of mice with sepsis (5). However, to the best of our knowledge, the protective effects of XBJ on HS have not been reported.

In the present study, the vital signs and survival times of rats with HS were observed, and the plasma levels of certain cytokines, biochemical indicators and coagulation indicators were detected. The mechanism by which XBJ protects against HS in rats was examined.

## Materials and methods

### Animals and main reagents

A total of 56 healthy adult male Sprague Dawley rats (clean grade; weight, 331–410 g; average weight, 376±23.6 g; provided by the Experimental Animal Center of the Chinese People’s Liberation Army General Hospital, Beijing, China) were included in this study. The rats were raised in cages (22±1°C, free access to food and water, 12-h light/dark cycle). XBJ was purchased from Tianjin Hongri Pharmaceutical Stock Co., Ltd. (Tianjin, China). [^125^I]-labeled tumor necrosis factor-α (TNF-α), interleukin (IL)-1β and IL-6 radioimmunoassay kits were provided by Beijing North Institute of Biological Technology (Beijing, China). This study was carried out in strict accordance with the recommendations in the Guide for the Care and Use of Laboratory Animals of the National Institutes of Health (4th edition, 2008). The animal use protocol was reviewed and approved by the Institutional Animal Care and Use Committee of the Chinese People’s Liberation Army General Hospital.

### Animal treatment

Eight rats were randomly selected for the establishment of animal models of HS in a preliminary experiment. The rats were exposed to a high temperature environment (42.5–43.5°C). The reduction of the mean arterial pressure (MAP) from the peak by 25 mmHg indicated the occurrence of HS (6). The time taken for HS to occur (t_HS_), the rectal temperature (Tr) and the heart rate (HR) were recorded.

Following anesthesia by intraperitoneal injection of 3% sodium pentobarbital (1 ml/kg), 24 rats were randomly divided into the normal control (NC), normal saline-treated HS (NS-HS) and XBJ-treated HS (XBJ-HS) groups, with eight rats in each group. In the NC group, the rats were placed in a 26°C environment and the Tr was maintained at 34°C, without any treatment. In the NS-HS group, prior to heat exposure, normal saline was injected into the femoral vein (4 ml/kg), followed by treatment with heat stress at 43°C for 47 min (in the preliminary experiment, the average t_HS_ was 46.88±1.25 min). Subsequently, the heat stress was removed and the rats were placed in a 26°C environment. In the XBJ-HS group, prior to heat exposure, XBJ was injected into the femoral vein (4 ml/kg), followed by treatment with heat stress at 43°C for 47 min. Subsequently, the heat stress was removed and the rats were placed in a 26°C environment. The MAP, Tr and HR were consecutively recorded and the changes in survival time (t_S_, from HS occurrence to death) were observed.

### XBJ effects on blood indicators and liver damage in the HS rats

Arterial blood (2.3 ml) was drawn at 0, 47 and 57 min after the initiation of the heat stress. The detection indices were as follows: i) After centrifugation at 4°C and 1,610 × g for 10 min, the serum was separated, and the concentrations of cytokine IL-1β, IL-6 and TNF-α were determined by radioimmunoassay (reagents were provided by Beijing North Institute of Biological Technology, Beijing, China); ii) the plasma levels of creatinine (Cr), blood urea nitrogen (BUN), aspartate aminotransferase (AST), alanine aminotransferase (ALT) and alkaline phosphatase (ALP) were determined by spectrophotometry (HITACHI7600; Hitachi High-Technologies, Tokyo, Japan); and iii) 0.9 ml arterial blood was combined with 0.1 ml 3.8% sodium citrate, was followed by centrifugation at 4°C and 716 × g for 7 min. The serum was separated, and the levels of activated partial thromboplastin time (APTT), prothrombin time (PT), fibrinogen degradation products (FDP) and D-dimer (D-D) were measured using CA-1500 automated coagulation instrument (SYSMEX Corporation, Kobe, Japan). Following the last blood drawing, the rats were sacrificed and three small sections of liver tissue (0.5×0.5×0.3 cm) were obtained. After fixation with neutral formalin, paraffin sections were prepared, followed by hematoxylin and eosin staining and observation under a XSP-10C light microscope (Shanghai Optical Instrument Factory, Shanghai, China).

### Statistical analysis

Data are expressed as the mean ± standard deviation. Statistical analysis was performed using SPSS statistical software, version 12.0 (SPSS, Inc., Chicago, IL, USA). The F-test was performed for analyzing the measurement data and the SNK-q test was used for multiple comparisons. P<0.05 was considered to indicate a statistically significant difference.

## Results

### Vital sign changes and t_HS_ of the HS rats

**Figure 1:**
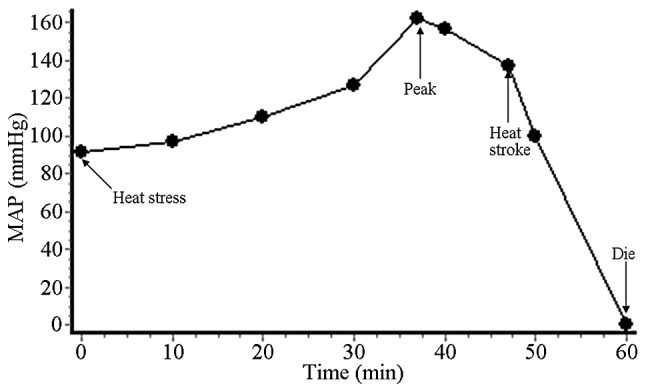
Effect of high-temperature heat stress on MAP (mmHg). MAP, mean arterial pressure; mmHg, millimeter of mercury.

Changes in the MAP under the high temperature environment (42.5–43.5°C) are shown in Fig. 1 and Table I. In the first 10 min of heat stress, the MAP did not significantly increase. In the subsequent 20 min, the rate of ascent of the MAP began to increase. From 30 to 37 min, the MAP quickly rose to a peak of ~160 mmHg. In the subsequent 10 min it declined to 136 mmHg, with a descent of 24 mmHg from the peak which indicated the occurrence of HS. The MAP at this moment was slightly higher than the basic level. In the final 13 min, the MAP quickly dropped to 0 mmHg and the rats died.

**Figure 2:**
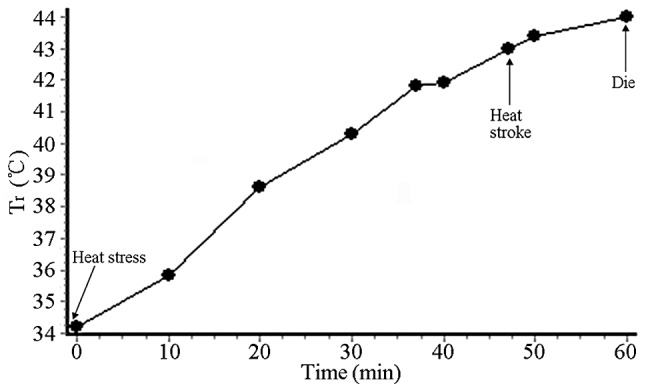
Effect of high-temperature heat stress on rectal temperature (Tr).

**Figure 3:**
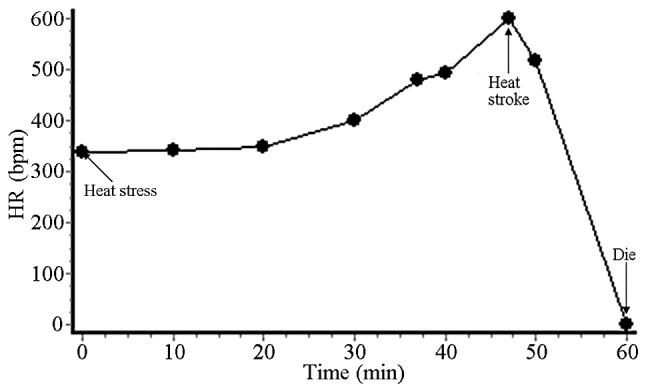
Effect of high-temperature heat stress on HR. HR, heart rate; bpm, beats per minute.

Fig. 2 shows the Tr changes in the HS rats. Following the initiation of the heat stress, the Tr presented a continuous, linear and rapid ascent. At the time of HS occurrence, the Tr reached 43.2°C. When the rats died, it was as high as 44°C. As shown in Fig. 3, in the first 20 min after the beginning of the heat stress, the HR was basically stable. In the subsequent 27 min, the HR rapidly rose and reached a peak [600 beats per minute (bpm)], with occurrence of HS. In the subsequent 13 min, heart arrhythmia appeared until the HR reached 0 bpm (the rats died). Under high-temperature heat stress in the preliminary experiment, the average t_HS_ was 46.875±1.246 min. Therefore, the t_HS_ in the following experiments was set as 47 min.

### Effects of XBJ on the vital signs and t_S_ of the HS rats

**Figure 4:**
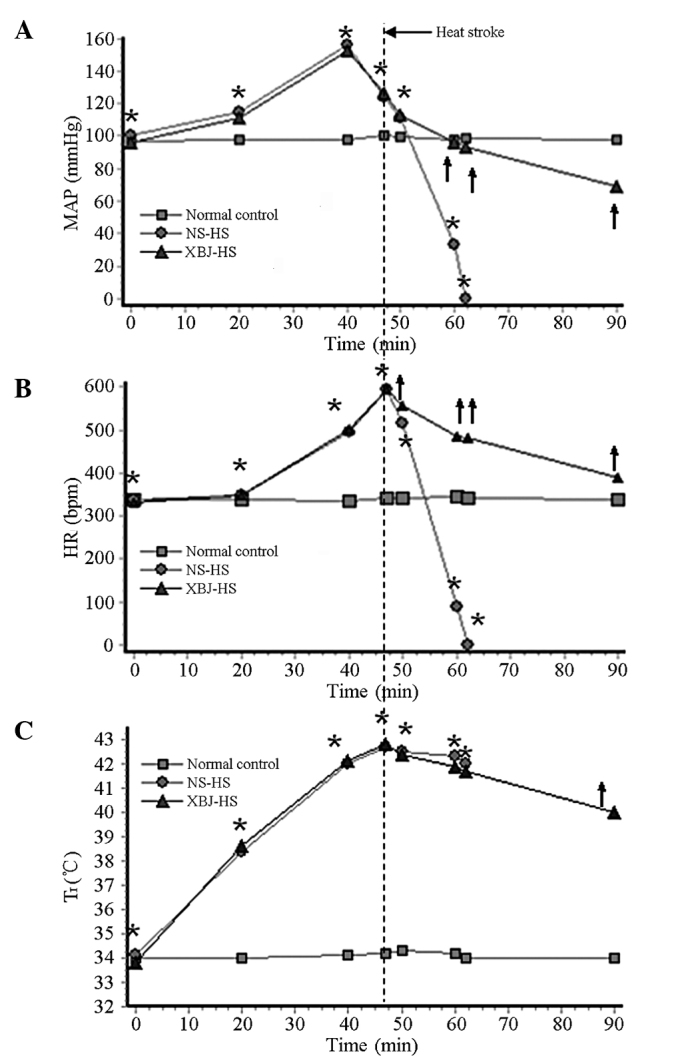
Effects of XBJ on the vital signs of HS rats. ^*^P<0.05 compared with the NC group; ^↑^P<0.05 compared with the NS-HS group. MAP, mean arterial pressure; mmHg, millimeter of mercury; NS, normal saline; HS, heat stroke; XBJ, Xuebijing injection; HR, heart rate; bpm, beats per minute; Tr, rectal temperature; NC, normal control.

Prior to the removal of heat stress, the trends of the changes of the vital signs in the NS-HS group were roughly the same as those in the XBJ-HS group. The MAP increased slowly in the first 20 min, then rapidly rose and reached a peak at 40 min, followed by a rapid reduction. The HR rose slowly in the first 20 min and then rapidly rose. At the HS time point, the HR reached a peak (600 bpm). The Tr gradually increased with the increase of heat stress time and reached 43°C at the HS time point. Following the removal of the heat stress, significant differences were identified in MAP and HR between the NS-HS and XBJ-HS groups. The MAP and HR in the NS-HS group decreased rapidly, and the rats died at 15 min after HS. In the XBJ-HS group, the MAP and HR decreased slowly. Following the removal of heat stress, the Tr did not markedly decrease in the NS-HS and XBJ-HS groups, and no significant differences were observed between the two groups (Fig. 4).

The effect of the heat stress on the t_S_ in the HS rats is shown in Table II. In the NC group, the rats were sacrificed by the intraperitoneal injection of a double dose of sodium pentobarbital at 480 min. The mean t_S_ in the XBJ-HS group was 74.625±4.627 min, which was significantly longer than that of the NS-HS group (15±2.07 min) (P<0.05).

### Effects of XBJ on blood indicators and liver damage in the HS rats

**Figure 5:**
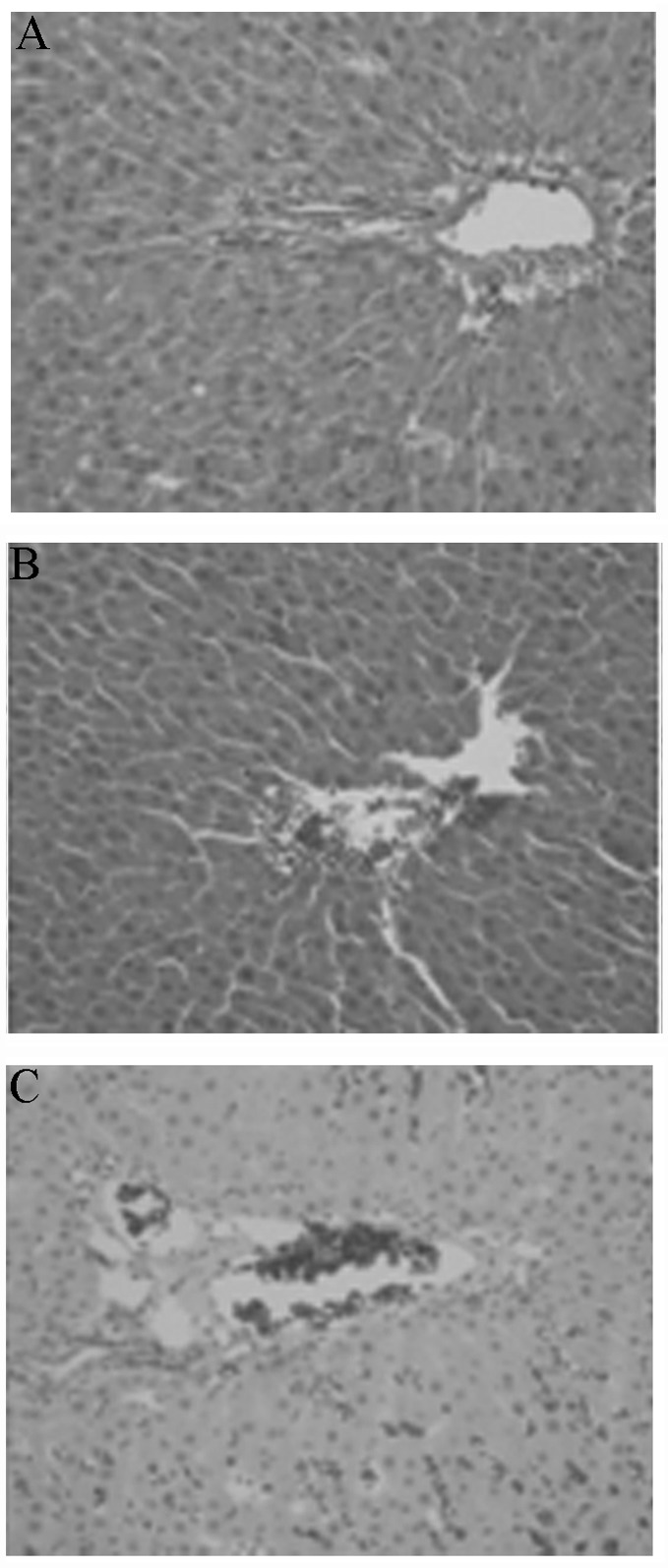
Liver tissue pathology results (magnification, ×40; staining, hematoxylin and eosin). (A) NC group; (B) XBJ-HS group; (C) NS-HS group. HS, heat stroke.

Tables III, IV and V show that at 47 min (the termination of heat stress) and 57 min, the levels of certain cytokines (IL-1β, IL-6 and TNF-α), coagulation indicators (APTT, PT, FDP and D-D) and biochemical indicators (Cr, BUN, AST, ALT and ALP) in the NS-HS group were significantly higher than those in the NC group (P<0.05). In the NS-HS group, the levels of the aforementioned indices were significantly reduced compared with those of the NC and NS-HS groups (P<0.05). Representative images of the liver tissue pathology results of each group are shown in Fig. 5. In the NS-HS group, serious liver cell congestion, nuclear swelling and central vein dilation were visible, along with the appearance of bubbles. In the XBJ-HS group, only a small number of congestive liver cells were identified, with occasional nuclear swelling but no bubbles, which was similar to that appearance of the NC group.

## Discussion

When rats are exposed to a high-temperature environment, the MAP, HR and Tr exhibit characteristic changes, along with the occurrence of HS. Under a high-temperature environment, a series of inflammatory cells are activated and they release large amounts of inflammatory cytokines, including IL-1, IL-2, IL-6, TNF-α and interferon, presenting a *‘*waterfall effect’. This can cause damage to body tissues and organs, which is similar to sepsis (2). Therefore, blocking this pathological link or decreasing the levels of inflammatory cytokines is key for the treatment of HS.

The results of the present study show that under a high temperature environment, rats present with tissue ischemia and damage (increases in the levels of Cr, BUN, AST, ALT and ALP), organ dysfunction (changes in the MAP and HR), hypercoagulable state or disseminated intravascular coagulation (DIC) (increases in the levels of APTT, PT, FDP and D-D) and an excessively activated systemic inflammatory response (increases in the levels of IL-1β, IL-6 and TNF-α). However, pretreatment with XBJ prior to the beginning of heat stress significantly inhibited the HS-induced systemic inflammatory response, tissue ischemia and damage, and organ dysfunction, thus extending the survival time of the rats.

XBJ is composed of safflower, radix *Paeoniae rubra*, radix *Salviae miltiorrhizae*, radix *Angelicae sinensis* and chuanxiong rhizome, of which the main effective components are safflor yellow A, ligustrazine, tanshinol, ferulic acid and paeoniflorin, respectively (7). Safflor yellow A dilates blood vessels, improves myocardial blood supply, reduces blood pressure, inhibits coagulation and thrombosis, reduces systemic hypoxia, increases tissue hypoxia tolerance and decreases capillary permeability (8). Ligustrazine markedly improves blood circulation, inhibits inducible nitric oxide synthase expression, reduces TNF-α levels and extends the survival time of rats with septic shock (9). Tanshinol promotes blood circulation and removes blood stasis, inhibits tissue ischemia-reperfusion injury, scavenges oxygen free radicals, protects mitochondria, regulates the thromboxane A2 (TXA_2_)/prostacyclin (PGI_2_) balance and immune response, and antagonizes endotoxins (10). Ferulic acid has a marked promoting effect on nonspecific, humoral and cellular immunity function (11). Angelica extract significantly inhibits high mobility group box 1 release and improves the survival rate of septic rats (12). Paeoniflorin improves heart and lung function, regulates the TXA_2_/PGI_2_ balance, inhibits platelet aggregation, prolongs thrombus formation time and prevents DIC. In addition, radix *P. rubra* significantly reduces plasma TNF-α levels and is effective in the treatment of sepsis (13). The combined effects of these components constitute the pharmacological basis of XBJ in the treatment of HS.

IL-1, IL-6 and TNF-α play crucial roles in the occurrence and development of HS (2). IL-1 is an endogenous pyrogen, which induces the inflammatory reaction in the acute period, with antitumor effects similar to those of tumor necrosis factors (14,15). It has been identified that the morbidity and mortality of HS are closely associated with endotoxemia and the release of IL-1 (16). Treatment with an IL-1 receptor antagonist (200 μg/kg) prior to HS occurrence weakens the cerebral ischemia and hypoxia of HS rats, prevents hypotension and prolongs the survival time (>600 min) (17,18). In a study by Chiu *et al* (18), following HS occurrence in rats, the continuous intravenous infusion of an IL-1 receptor antagonist (200 μg/kg·h) was immediately performed. The results showed that the level of dopamine released by the brain was reduced from 275% in rats with untreated HS to 140%, with a significantly prolonged survival time (>600 min) (18). IL-6 is highly correlated with the mortality of HS and neurological symptoms. IL-6 antagonists are likely to become a novel breakthrough for the prevention and treatment of HS (6). As demonstrated in a primate animal model of HS, the concentration of IL-6 is associated with the severity of HS (19). TNF-α induces fever, stimulates white blood cells and neutralizes HSPs (1). High levels of TNF-α cause an excessive inflammatory response, increased vascular permeability, hemodynamic disorders, microcirculatory disturbances and cell dysfunction, leading to MODS which is associated with the disease severity and prognosis (14). In the present study, the plasma levels of TNF-α, IL-1β and IL-6 in the NS-HS group were significantly increased compared with those in the control group, and the extents of the increases of these indices were significantly reduced by XBJ. This indicates that XBJ reduced the secretion of inflammatory cytokines, which should be one of the important anti-endotoxin mechanisms for HS. A study has identified that XBJ reduces the levels of reperfusion injury of intestinal mucosa, protects the intestinal mucosal barrier function and prevents the invasion of intestinal endotoxins into the blood, thereby reducing the release of inflammatory cytokines (20).

HS is one manifestation of the inflammatory and anti-inflammatory response under high temperature conditions. An excessively activated inflammatory response and DIC are the main factors leading to the mortality of patients and deaths of animals with HS (21). In patients with HS, coagulation system disorders and a runaway inflammatory response are very common and are closely associated with the disease severity and prognosis (22). It has been confirmed in clinical and experimental studies that XBJ is not only able to inhibit the coagulation/anticoagulation imbalance and the release of harmful vasoactive mediators, but also blocks the trigger factors for coagulation function disorders (23–26). The results of the present study demonstrate that XBJ significantly reduces the APTT and PT, and the plasma concentrations of FDP and D-D, and that it has a protective effect on blood coagulation function.

At present, lowering the body temperature is one of the main methods for the treatment of patients with HS. It has been confirmed in numerous studies that hypothermia therapy significantly inhibits cerebrovascular dysfunction, the systemic inflammatory response, hypercoagulable state or DIC, cerebral oxidative stress, and ischemia and injury in patients with HS (27–29). In the present study, no specific efforts to lower the body temperatures of the rats were made, and pretreatment with XBJ was performed prior to the application of heat stress. The results show that XBJ significantly reduces HS-induced circulation dysfunction, hypercoagulability or DIC, and tissue ischemia and injury, but it does not reduce the body temperature of HS rats. This indicates that high fever is not the only pathogenic factor for HS, and XBJ exerts therapeutic effects by inhibiting the inflammatory response and improving coagulation function.

## Figures and Tables

**Table I:** Time taken for HS to occur and vital signs at the time of HS occurrence.

Rat	t_HS_ (min)	MAP_HS_ (mmHg)	T_rHS_ (°C)	HR_HS_ (bpm)
1	46	141	42.8	605
2	48	131	43.4	580
3	47	129	43.0	595
4	45	141	42.8	606
5	47	143	43.6	616
6	49	134	42.9	578
7	47	137	42.4	591
8	46	138	43.0	599
Mean ± SD	46.875±1.246	136.75±5.036	42.987±0.372	596.25±13.069

t_HS_, time taken for HS to occur; HS, heat stroke; MAP_HS_, mean arterial pressure at HS occurrence; mmHg, millimeter of mercury; T_rHS_, temperature at HS occurrence; HR_HS_, heart rate at HS occurrence; bpm, beats per minute.

**Table II:** Effects of XBJ on the survival time of HS rats.

Rat	NS-HS	XBJ-HS	NC
1	11	75	480
2	15	68	480
3	13	80	480
4	17	73	480
5	16	69	480
6	16	75	480
7	15	76	480
8	17	81	480
Mean ± SD	15±2.07^*^	74.625±4.627^a^,^b^	480

a P<0.05 compared with the NC group;

b P<0.05 compared with the NS-HS group

XBJ, Xuebijing injection; t_s_, survival time from HS occurrence; HS, heat stroke; NS, normal saline; NC, normal control.

**Table III:** Effects of XBJ on certain cytokines in HS rats.

Group	Heat stress time (min)	IL-1β (ng/ml)	IL-6 (pg/ml)	TNF-α (ng/ml)
NC	0	0.315±0.040	89.054±6.061	0.991±0.133
	47	0.305±0.042	93.685±3.841	0.940±0.076
	57	0.314±0.036	94.633±4.697	0.966±0.065
NS-HS	0	0.288±0.024	90.850±4.227	1.011±0.096
	47	1.246±0.090^a^	389.482±18.904^a^	5.620±0.321^a^
	57	2.547±0.146^a^	494.754±18.249^a^	5.867±0.212^a^
XBJ-HS	0	0.305±0.042	91.164±4.339	0.979±0.085
	47	0.686±0.069^a^,^b^	192.679±11.568^a^,^b^	2.620±0.321^a^,^b^
	57	0.710±0.046^a^,^b^	209.912±9.779^a^,^b^	2.503±0.261^a^,^b^

a P<0.05 compared with the NC group;

b P<0.05 compared with the NS-HS group.

XBJ, Xuebijing injection; HS, heat stroke; NC, normal control; NS, normal saline; IL, interleukin; TNF-α, tumor necrosis factor-α.

**Table IV:** Effects of XBJ on certain coagulation indicators in HS rats.

Group	Heat stress time (min)	APTT (sec)	FDP (mg/l)	D-D (μg/ml)	PT (sec)
NC	0	23.887±1.476	172.375±7.009	45.563±1.868	15.800±0.338
	47	24.225±1.419	169.500±7.211	45.250±2.112	15.950±0.434
	57	24.075±1.492	173.750±8.172	45.425±2.218	15.850±0.302
NS-HS	0	23.100±1.533	167.250±7.760	46.375±2.000	15.762±0.233
	47	69.362±7.751^a^	251.375±8.501^a^	85.850±3.113^a^	20.213±0.340^a^
	57	89.975±7.674^a^	281.875±8.593^a^	112.85±3.275^a^	23.575±0.212^a^
XBJ-HS	0	23.938±1.814	168.625±8.280	45.588±2.149	15.830±0.282
	47	45.962±5.756^a^,^b^	199.750±5.994^a^,^b^	78.100±3.106^a^,^b^	16.988±0.368^a^,^b^
	57	48.725±4.642^a^,^b^	215.750±6.840^a^,^b^	81.775±3.397^a^,^b^	17.587±0.300^a^,^b^

a P<0.05 compared with the NC group;

b P<0.05 compared with the NS-HS group.

XBJ, Xuebijing injection; HS, heat stroke; NC, normal control; NS, normal saline; APTT, activated partial thromboplastin time; FDP, fibrinogen degradation products; D-D, D-dimer; PT, prothrombin time.

**Table V:** Effects of XBJ on certain biochemical indicators in HS rats.

Group	Heat stress time (min)	Cr (mg/dl)	BUN (mg/dl)	AST (U/l)	ALT (U/l)	ALP (U/l)
NC	0	18.825±1.930	6.031±1.014	101.263±11.696	45.475±5.393	196.388±20.508
	47	20.125±1.807	6.106±0.588	100.962±11.924	43.213±3.991	196.688±14.283
	57	20.900±1.373	6.095±0.353	96.575±12.115	43.338±6.265	198.175±12.924
NS-HS	0	21.025±2.696	6.649±0.671	97.862±13.362	42.525±4.134	200.025±11.366
	47	42.438±1.846^a^	21.696±1.419^a^	376.262±14.516^a^	140.750±8.665^a^	348.212±8.136^a^
	57	67.412±2.106^a^	27.851±0.593^a^	474.238±15.365^a^	168.787±6.906^a^	428.500±21.758^a^
XBJ-HS	0	20.263±1.694	6.661±0.586	100.938±10.913	44.587±4.637	199.450±13.010
	47	30.788±1.398^a^,^b^	14.335±0.667^a^,^b^	220.838±22.345^a^,^b^	70.888±7.578^a^,^b^	202.825±9.102^a^,^b^
	57	36.100±1.704^a^,^b^	17.821±1.093^a^,^b^	265.800±17.029^a^,^b^	84.000±10.366^a^,^b^	207.637±11.282^a^,^b^

a P<0.05 compared with the NC group;

b P<0.05 compared with the NS-HS group.

XBJ, Xuebijing injection; HS, heat stroke; NC, normal control; NS, normal saline; Cr, creatinine; BUN, blood urea nitrogen; AST, aspartate aminotransferase; ALT, alanine aminotransferase; ALP, alkaline phosphatase.
